# AraLeTA: An Arabidopsis leaf expression atlas across diurnal and developmental scales

**DOI:** 10.1093/plphys/kiae117

**Published:** 2024-03-01

**Authors:** Gina Y W Vong, Kayla McCarthy, Will Claydon, Seth J Davis, Ethan J Redmond, Daphne Ezer

**Affiliations:** Department of Biology, University of York, York YO10 5DD, UK; Department of Biology, University of York, York YO10 5DD, UK; Department of Biology, University of York, York YO10 5DD, UK; Department of Biology, University of York, York YO10 5DD, UK; Department of Biology, University of York, York YO10 5DD, UK; Department of Biology, University of York, York YO10 5DD, UK

## Abstract

Mature plant leaves are a composite of distinct cell types, including epidermal, mesophyll, and vascular cells. Notably, the proportion of these cells and the relative transcript concentrations within different cell types may change over time. While gene expression data at a single-cell level can provide cell-type-specific expression values, it is often too expensive to obtain these data for high-resolution time series. Although bulk RNA-seq can be performed in a high-resolution time series, RNA-seq using whole leaves measures average gene expression values across all cell types in each sample. In this study, we combined single-cell RNA-seq data with time-series data from whole leaves to assemble an atlas of cell-type-specific changes in gene expression over time for Arabidopsis (*Arabidopsis thaliana*). We inferred how the relative transcript concentrations of different cell types vary across diurnal and developmental timescales. Importantly, this analysis revealed 3 subgroups of mesophyll cells with distinct temporal profiles of expression. Finally, we developed tissue-specific gene networks that form a community resource: an Arabidopsis Leaf Time-dependent Atlas (AraLeTa). This allows users to extract gene networks that are confirmed by transcription factor–binding data and specific to certain cell types at certain times of day and at certain developmental stages. AraLeTa is available at https://regulatorynet.shinyapps.io/araleta/.

## Teaser

An atlas of cell-type-specific changes in gene expression over time enables the extraction of gene networks associated with certain cell types at specific times.

## Introduction

The coordination of spatial and temporal gene expression dynamics is fundamental to plant development and response to environmental stimuli. Organisms have distinct gene regulatory programs within different cell types, which regulate the changes in gene expression over diurnal (Yakir et al. 2011) and developmental timescales (Ma et al. 2005). Each of these regulatory programs coordinates the changes in gene expression over time to respond to both intrinsic (time of day and maturity) and extrinsic factors (environmental stimuli) (Sheen 1994). It is the coordination of transcriptional patterns in discrete cell types that provides determinate capacity for cell function in a context of its tissue and organ.

Multiple approaches have been used to measure gene expression over space and time, but each has its drawbacks. Through live image–based assays, it is possible to track gene expression of a small number of genes over both time and space (Shav-Tal et al. 2004). For instance, Gould et al. (2018) identified waves of circadian gene expression originating from the meristems. In contrast, RNA-seq enables researchers to measure gene expression of all mRNAs at once. Many researchers perform high temporal resolution RNA-seq time-series experiments to infer how gene expression changes over time (e.g. Krouk et al. 2010; Cortijo et al. 2017; Ezer and Keir 2019; Balcerowicz et al. 2021). However, these studies do not capture the cell-type-specific changes in gene expression. Additionally, over 80% of leaf expression in a bulk RNA-seq sample originates from mesophyll cells, which will mask gene expression patterns from other cell types, such as in the vasculature and epidermis tissues (Endo et al. 2014). Bulk RNA-seq also masks heterogeneity within mesophyll cell populations (Procko et al. 2022). Increasingly, single-cell and tissue-specific RNA-seq have been used to characterize cell-type-specific gene expression patterns in leaf (Liu et al. 2020; Kim et al. 2021; Lopez-Anido et al. 2021), root (Shahan et al. 2022), and meristem (Neumann et al. 2022) tissue, but it is prohibitively expensive to perform these in a high-resolution time series. Lee et al. (2023) have developed a developmental single-cell atlas, but it covers only 5 vegetative stages and is therefore not at a similar temporal resolution as existing bulk RNA-seq resources and would be too expensive to replicate under a wide range of experimental conditions. Recent efforts are underway to construct further Plant Cell Atlases based on single-cell analysis (Plant Cell Atlas Consortium et al. 2021), and it is important to find methods to best utilize these kinds of resources, especially when investigating processes that occur over time.

Our work here demonstrates the potential of integrating single-cell RNA-seq data and high-resolution time-series data to unravel cell-type-specific gene networks in Arabidopsis (*Arabidopsis thaliana*). Drawing inspiration from cancer cell dynamics research (Newman et al. 2019), we used CIBERSORTx, a powerful technique that combines single-cell RNA-seq and bulk RNA-seq data (Newman et al. 2019) and that outperforms other deconvolution methods in a large benchmarking study (Sutton et al. 2022). We inferred relative expression in various cell types across samples and estimated cell-type-specific gene expression values. By applying these techniques, we gained insights into the dynamics of expression within different cell types across varying temporal scales. Although Procko et al. (2022) identified 4 subpopulations of mesophyll with unclear distinguishing markers, our analysis revealed changes in their relative expression levels over diurnal and developmental scales, raising questions about cell-state changes and activity-level variations over time, as well as the relative light sensitivity of different mesophyll cell states. Moreover, differences in the relative expression of mesophyll subgroups between bolted and unbolted plants were observed. By incorporating transcription factor–binding data (O’Malley et al. 2016), we have provided a valuable resource for the Arabidopsis community: a leaf cell–type network available at https://regulatorynet.shinyapps.io/araleta/. We also highlight relevant portions of this network during diurnal and developmental timescales.

## Results

### Detection of cell-type transcriptional activity in bulk RNA-seq by utilizing single-cell RNA-seq data

**Figure 1.**
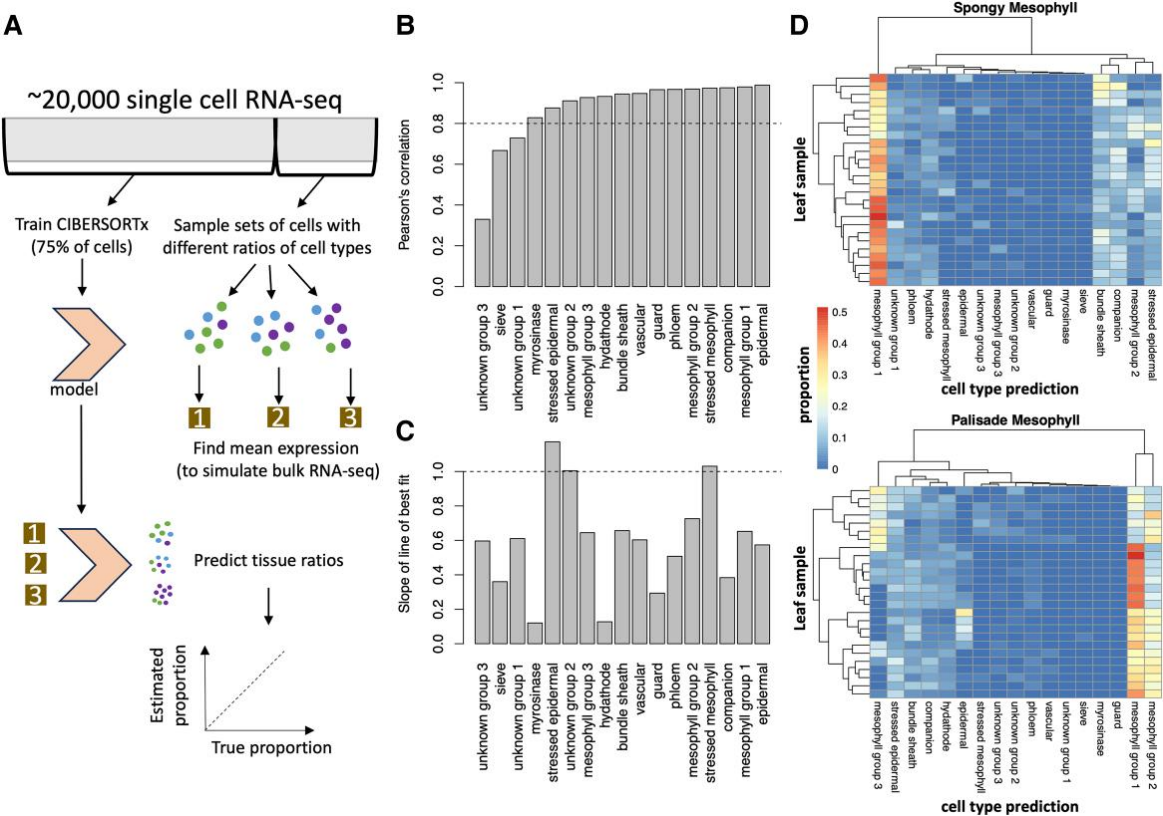
CIBERSORTx predictions for simulated bulk RNA-seq samples. **A)** Seventy-five percent of cells per cluster were used to train a CIBERSORTx model, while the remaining 25% of cells were repeatedly subsampled to generate 500 simulated bulk RNA-seq samples with mixed cell types (see the Materials and methods section). **B)** Pearson's correlation between the true cell-type proportions in the simulated bulk RNA-seq sample and the tissue proportions predicted by CIBERSORTx. All the correlations were statistically significant (*P* < 0.05), with Pearson's correlations >0.8 highlighted by the horizontal bar. **C)** The slope of the line of best fit for a comparison between the predicted and the true cell-type proportions (scatterplots in Supplementary Fig. S2). Values close to 1 (horizontal line) are most accurate. Taken together, these results suggest that CIBERSORTx can predict the relative proportions within the same cell type between RNA-seq samples, but that it consistently overpredicts or underpredicts certain cell types within an RNA-seq sample. **D)** Predicted cell-type proportions in microdissected leaf samples, with each row representing a leaf and each column representing a different predicted cell type. Note that phloem is short for phloem parenchyma.

First, we wish to confirm that we can accurately predict proportions of cell types in bulk RNA-seq samples using single-cell RNA-seq data from *A. thaliana* by training CIBERSORTx on a training set of single-cell RNA-seq cells and then testing its accuracy on deconvolving simulated bulk RNA-seq samples constructed from subsets of the remaining cells (Fig. 1A). When we simulated bulk RNA samples containing a single-cell type, we correctly identified all tissue types, except for hydathodes, as these were often misclassified as mesophyll cells (Supplementary Fig. S1, A and B). We chose to use Procko et al. (2022) because it had a larger number of leaf cells than many other single-cell datasets, and therefore, we were able to include rarer cell types. Moreover, the authors did not artificially enrich their selection of specific cell types, and therefore the ratio of cell types would be roughly similar to that found in natural leaves. In this paper, we named the 3 mesophyll cell clusters (specifically, Clusters 1, 3, and 4) from Procko et al. (2022) as mesophyll Groups 1, 2, and 3, respectively. We also named the 3 clusters of unknown type (termed Clusters 10, 11, and 16) as unknown Groups 1, 2, and 3, respectively (Supplementary Fig. S1A).

Next, we simulated bulk RNA-seq samples with mixed cell types. We accurately predicted the relative abundance of simulated cell types between these samples (Supplementary Table S1), with Pearson's *R* >0.8 for all cell types except for 2 unidentified cell types and sieve cells (Supplementary Fig. S2, Fig. 1B). However, we note that CIBERSORTx is best at predicting the relative amounts of cell types between samples, rather than the relative proportions of cell types within a sample—a known issue with gene expression deconvolution algorithms (Sutton et al. 2022). It consistently overestimated the proportion of stressed cells and underestimated the proportion of other cell types (Fig. 1C). These results confirm that CIBERSORTx, a technique initially developed for mammalian research (Newman et al. 2019), can be applied to plant systems.

CIBERSORTx was also successful at predicting the tissue composition of simulated bulk RNA-seq samples generated from other single-cell RNA-seq experiments (Fig. 1D, Supplementary Fig. S3). We chose to test our model on cells in Xia et al. (2022) as these were isolated using microdissection, so that we would be able to know the spatial localization of these cells. In contrast, in marker-based selection, there is always a risk that the marker is also expressed in an off-target cell type. Interestingly, microdissected spongy mesophyll cells from Xia et al. (2022) tended to be classified as mesophyll cells from Group 1, while the palisade cells were more evenly split among the 3 mesophyll groups that Procko et al. (2022) previously reported (Fig. 1D). This suggested that atlases of single-cell RNA-seq expression can serve as reusable resources in the community for estimating cell-type compositions of bulk samples.

Next, we explored the parameters of the signature matrix constructed from the training set to help with deconvolving the bulk RNA-seq samples. CIBERSORTx selected 4950 genes for use in predicting the cell-type proportion (Supplementary Fig. S4), and these have significant enrichment (*P* < 0.01) for Gene Ontology (GO) terms associated with ion binding, catalytic activity, structural constituents of chromatin, transmembrane transporter activity, response to stimulus/stress, and amino acid metabolic processes (see Supplementary Table S2 [signature matrix] and Supplementary Table S3 [gProfiler outputs]). These cell-type-specific processes appear to be capable of distinguishing cell-type proportions.

### Across developmental scales, transcriptional activity shifts from epidermal to vascular cell types

We next sought to identify how cell-type transcriptional activities changed over different temporal scales. For this, we applied the signature matrices that we previously inferred from single-cell RNA-seq data to interpret bulk RNA-seq time-series datasets. It is important to note that deconvolution algorithms like CIBERSORTx do not find the proportion of cells of each type but rather the proportion of transcripts that is attributable to different cell types. Changes in cell-type proportion over time could be attributable to changes in the total number of cells at a certain time, increased activity of certain cell types, or cells changing their expression profile to mimic the expression of a different cell type. For conciseness, we will refer to the output of CIBERSORTx as the “activity” of specific cell types.

**Figure 2.**
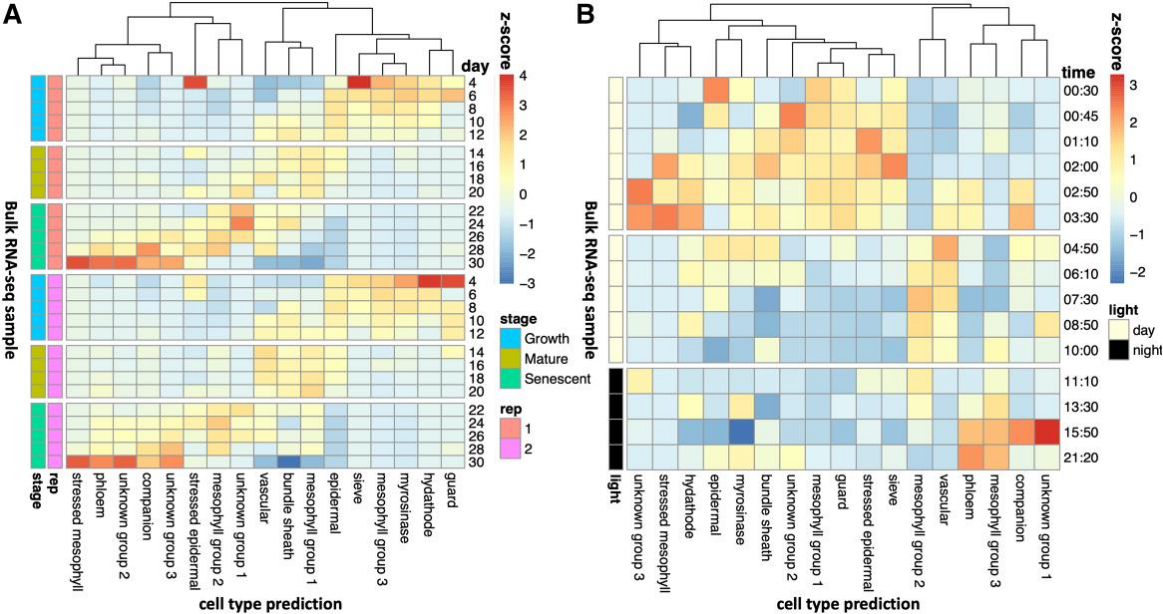
Shifts in cell-type proportions over developmental and diurnal time series. We predicted the proportion of cell types in a **A**) developmental time series (Woo et al. 2016) and **B**) diurnal time series (Hickman et al. 2017), utilizing a reference leaf scRNA-seq dataset (Procko et al. 2022). In **A**), each row represents a different RNA-Seq sample, with the proportions normalized using *z*-scores. The developmental stage and the biological replicate are indicated by the colored bars. In **B**), the proportions of cell types were predicted independently for each of the 4 biological replicates, and then these were averaged for each row and normalized by *z*-scores. The times are relative to dawn.

Next, we investigated whether the proportion of cell type expression levels varied across a developmental timescale (Fig. 2A, Supplementary Fig. S5A and Table S4), utilizing a leaf developmental time-series RNA-seq dataset (Woo et al. 2016). The reference single-cell RNA-seq experiment was performed on the first true leaves at 17 d postgermination, and therefore, it only captures cell-type-specific transcriptional activity at a single snapshot. During the growth-to-senescence transition, we detected a decrease in epidermal expression and an increase in vascular expression. Rarer cell types, like guard cells and hydathodes, seemed to have a higher proportion early in development, possibly because these cell types get diluted as the leaf expands (Ietswaart et al. 2017) or because these rarer cell types have gene expression profiles that mimic other cell types when mature, as previously shown for guard cells (Adrian et al. 2015). Phloem parenchyma expression and stressed mesophyll expression were maximized in the senescing leaves compared with other developmental stages. This is consistent with phloem parenchyma cells contributing to nutrient redistribution in the senescing leaf and with the transformation of neighboring cell types into phloem cells during senescence (Hunziker et al. 2019). Although there is currently no leaf-specific developmental single-cell atlas to compare our results with, Lee et al. (2023) performed a whole rosette single-cell RNA-seq in 3 developmental timepoints. Both this dataset and CIBERSORTx show a decline in bundle sheath and vascular cells from Day 17 onward. Lee et al. (2023) showed that the proportion of epidermal cells stays stable, as the plant transitions to senescence, suggesting that our observed decline in epidermal cells may reflect a change in transcriptional activity, rather than a decline in cell count. Our results contradict Lee et al. (2023) in terms of phloem parenchyma and companion cell expression, which we predict increase and Lee et al. (2023) predict decline. These discrepancies may result from the activity of phloem parenchyma cells in nonleaf tissue in the Lee et al. (2023) dataset, as it includes whole rosettes. Moreover, Lee et al. (2023) appeared to have a larger proportion of senescing cell types in Day 17 rosettes than at older ages, and therefore, the sampled rosettes at this timepoint may have been stressed, making direct comparisons challenging.

Intriguingly, the 3 groups of nonstressed mesophyll cells also displayed distinct temporal patterns. Group 3 mesophylls had peak expression during the growth phase, Group 1 mesophylls had peak expression at the mature leaf phase, and Group 2 mesophylls had peak expression during the mature-leaf-to-senescence transition period. Group 1 cells were found most frequently in the leaves, as in Procko et al. (2022), which is consistent with their sample collection timepoint of 17 d. This analysis suggests that mesophyll-specific expression profiles transition among the 3 different states identified by Procko et al. (2022) over developmental timescales.

### Diurnal oscillations of mesophyll, epidermal, vascular, and phloem transcriptional activity

Additionally, we were interested in how the cell-type-specific gene expression patterns varied across diurnal timescales, as measured in a leaf diurnal time-series RNA-seq dataset (Hickman et al. 2017; see Fig. 2B, Supplementary Figs. S5B, S6, and Table S5). Procko et al. (2022) grew their leaves under continuous light to minimize the impacts of the clock, but plant clocks are synchronized by the initial seed imbibement and so they will still experience consistent daily oscillations (Zhong et al. 1998). Even if averages of transcripts appear “arrhythmic” after prolonged plant acclimation to constant conditions, the cells that pattern tissues are still robustly rhythmic, although asynchronous from each other (Yakir et al. 2011; Gould et al. 2018). Thus, the individual cells sampled by Procko et al. (2022) will each be in an unknown phase of the circadian clock.

Epidermal expression is maximized in the morning. Meanwhile, vascular expression peaks in the afternoon. We hypothesize that this is when the plant is most water-stressed, due to the tendency for this time of day to have a higher temperature. Phloem parenchyma expression peaks at the end of the night when passive loading of sugars through plasmodesmata is replaced with active apoplasmic loading (Wei et al. 2021). Interestingly, epidermal stress cell expression peaks during the ZT1 dawn burst of expression (Balcerowicz et al. 2021), while mesophyll stress expression peaks a few hours afterward (ZT2-4). One hypothesis is that epidermal cells become stressed by the lights suddenly turning on within the growth cabinet, while mesophyll cells become stressed because of reactive oxygen species accumulation as a result of photosynthesis.

The 3 nonstressed mesophyll groups primarily have peak expression at different times of the day. The mesophyll cells that are predicted to be active during early development (Group 3) are also predicted to be active late at night. The mesophyll cells that were active in mature leaves (Group 1) were also active in the morning. A comparison with Xia et al. (2022) suggests that this mesophyll group may be enriched for spongy mesophyll cells. The mesophyll group that was most active during the transition to senescence (Group 2) was also active during the afternoon and early evening. The mesophyll subgroups each appear to have distinct temporal transcriptional profiles, both on a diurnal scale and on developmental scale.

### Expression of time-of-day–dependent light-sensitive genes across cell types

**Figure 3.**
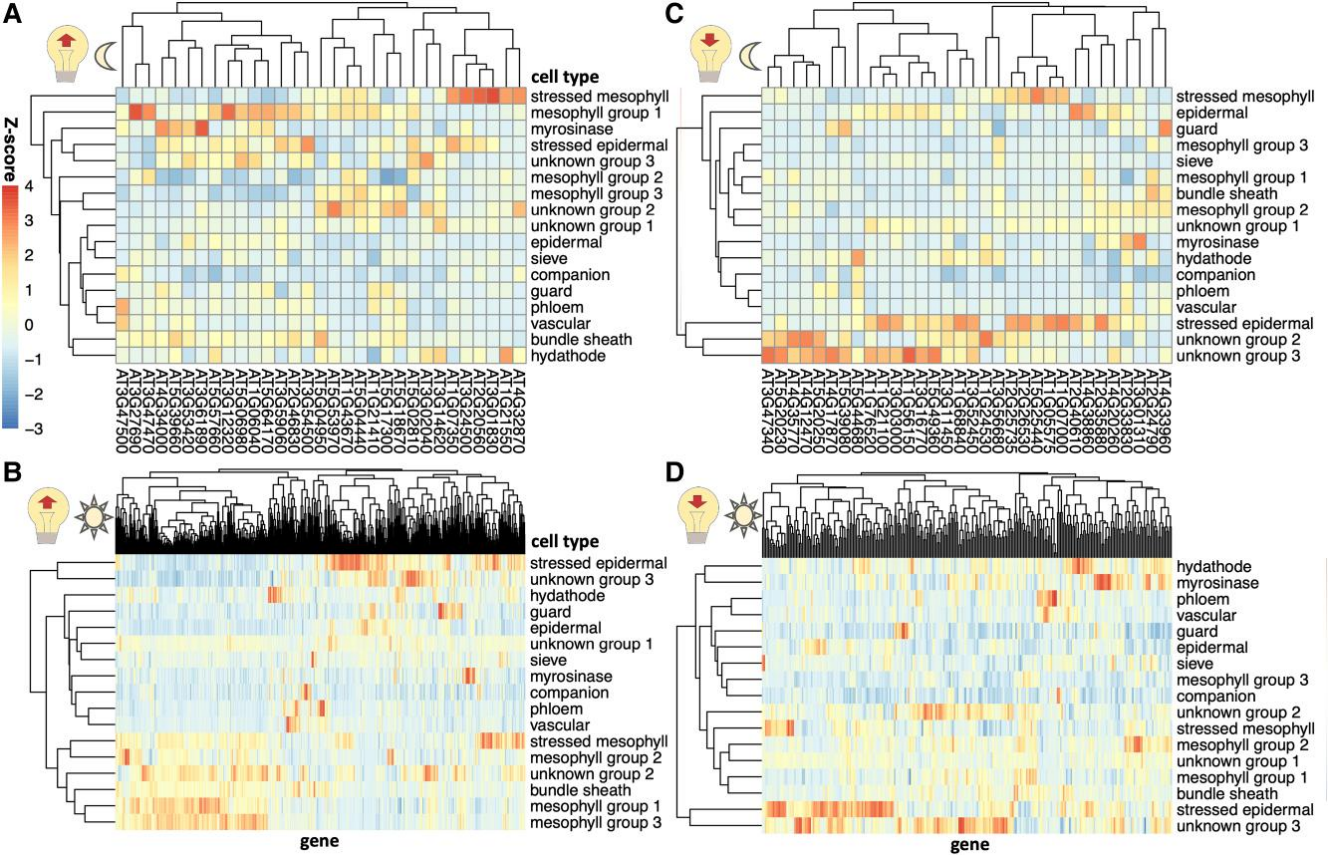
The expression of light-sensitive genes in single-cell leaf data. Sets of genes were selected that are light-induced **A)** and **B)** or light-repressed **C**) and **D)** during either a nocturnal light treatment **A**) and **C)** or a light treatment after an extended night **B**) and **D)**, based on Rugnone et al. (2013). The mean expression of these genes in cells in each cluster in Procko et al. (2022) was calculated, and *z*-scores were calculated for each column.

Next, we evaluated whether the cell types had different levels of expression of light-responsive genes. We hypothesized that Group 1 mesophyll cells may include more of the genes that are induced by light exposure at the end of the night, as these cells are most active in the morning. To evaluate this hypothesis, we analyzed the expression of light-induced genes (Rugnone et al. 2013) in the Procko et al. (2022) single-cell dataset. Consistent with our hypothesis, genes that are induced by light exposure at night (Fig. 3A) tended to be found in mesophyll Group 1 (morning/mature phase mesophyll group). This set of mesophyll Group 1 genes included genes encoding NIGHT LIGHT–INDUCIBLE AND CLOCK-REGULATED1,3 (LNK1, LNK3), which help entrain the circadian clock in response to light in the morning (Xie et al. 2014), SALT TOLERANCE (STO), which is a BBX family protein involved in the morning dawn burst of expression (Balcerowicz et al. 2021), and members of the light-harvesting complex in photosynthesis (LHCB2.4, LHCA4). Thus, mesophyll clusters indeed harbored notable light-induced genes.

A different set of genes that are light-sensitive at night were more highly expressed in stressed mesophyll cells. These included genes encoding DNAJ, a heat shock protein (Pulido and Leister 2017), SERINE/ARGININE RICH-LIKE PROTEIN 45A (SR45a), a stress-induced splicing factor that regulates anthocyanin accumulation (Gulledge et al. 2012; Albaqami 2023), and MULTIPROTEIN BRIDGING FACTOR 1C (MBF1C), whose expression is elevated in response to a wide range of stresses (Lee and Bailey-Serres 2019). These results suggest that light exposure earlier than anticipated may induce a light stress response, in addition to activating morning mesophyll expression, and that these 2 responses impact the activity of 2 different sets of genes in 2 different cell types. In contrast, genes that are induced by light exposure after an extended night (Fig. 3B) are primarily expressed either in the mesophyll or in stressed epidermal cells.

Despite the plants in Procko et al. (2022) being exposed to continuous light, some cell types continue to express genes that are repressed under light exposure. Specifically, unknown Groups 2 and 3 contain genes that have decreased expression under light exposure at night like *SENESCENCE1* (*SEN1*), *DARK INDUCIBLE 10* (*DIN10*), and *GLUTAMINE-DEPENDENT ASPARAGINE SYNTHASE 1* (*ASN1*), which are all also induced by senescence (Fujiki et al. 2001). Coupled with our previous observations that their activity peaks during late senescence (Fig. 2A), we propose that these cell types are associated with senescence. Stressed epidermal cells also contain high levels of light-repressed genes. Genes that have reduced expression under light exposure are not expressed highly in the cell types that are predominantly found enriched during the night (phloem, mesophyll Group 3, and companion cells, Fig. 2B), but this may be because the scRNA-seq was performed in plants under continuous light (Fig. 3, C and D). This suggests that it may be wise to perform scRNA-seq on more realistic diurnal conditions, sampling multiple times a day, to adequately capture the cell-specific transcription over the course of a day.

### Cell-type-specific regulatory program during bolting

**Figure 4.**
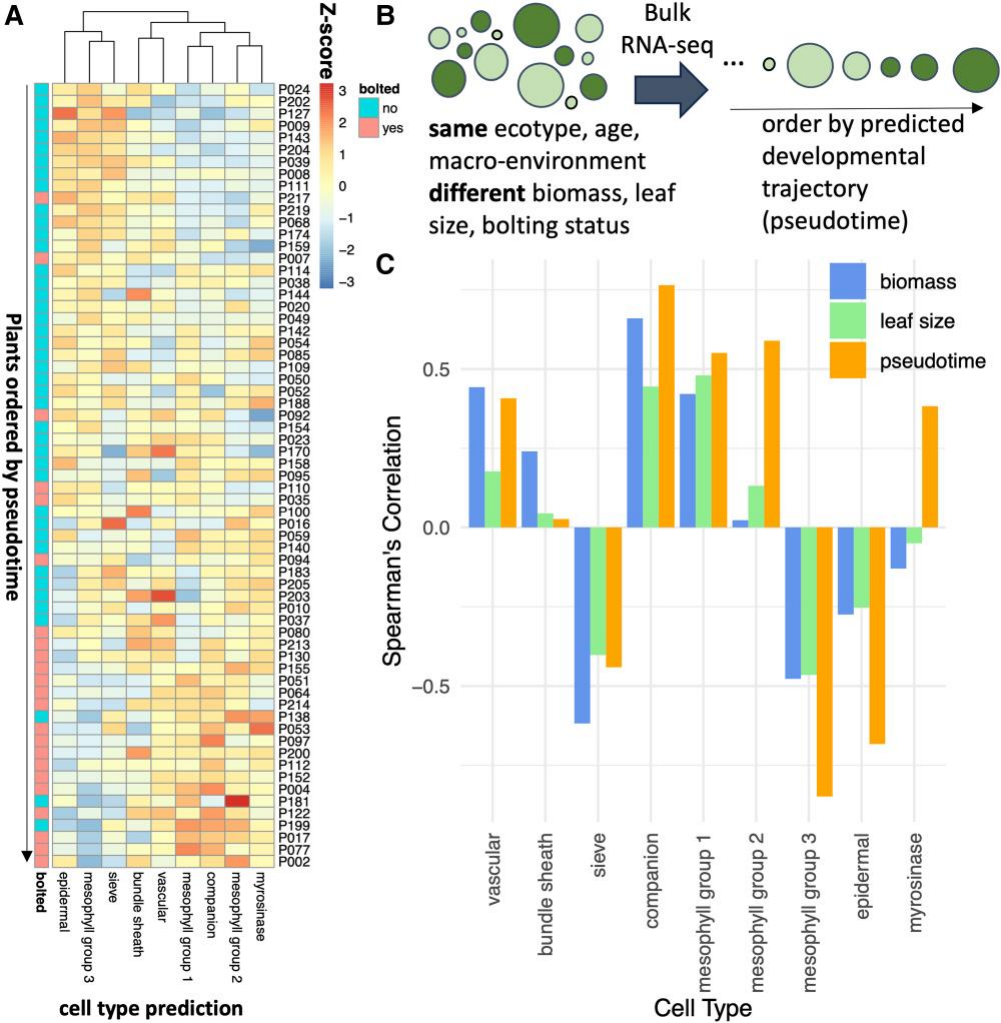
Cell activity changes during bolting. **A**) We predicted the proportion of cell types in plants immediately before and after bolting (Redmond et al. 2023), utilizing a reference leaf scRNA-seq dataset (Procko et al. 2022). Each row represents a different RNA-seq sample (representing a single plant), with the proportions normalized using *z*-scores. **B)** The plants in **A**) are ordered by pseudotime, which was calculated by Redmond et al. (2023) as an arrangement of 65 individual plants sampled along a developmental trajectory. **C)** For each cell type, the Spearman ranked correlation was calculated between the proportion of that cell type in each plant versus another trait of the plant (biomass, leaf size, or pseudotime).

We have observed that there are differences in the transcriptional activity of cell types across a developmental and a diurnal timescale. Next, we decided to focus on the changes that happen during a rapid developmental transition, specifically bolting, which coincides with the start of senescence (Redmond et al. 2023). Mimicking the pattern observed during the developmental time series (Fig. 2A), the relative expression of mesophyll Group 3 (the growth-phase mesophyll) went down, while the expression of mesophyll Groups 1 and 2 (the mature phase and senescence mesophyll) went up over pseudotime (Fig. 4A, Supplementary Fig. S7, A and B and Table S6), where pseudotime refers to the predicted ordering of individual plants in Redmond et al. (2023) over a developmental trajectory on the basis of bulk RNA-seq data (Fig. 4B, Supplementary Table S6). Also consistent with the developmental time series, we observe a decrease in epidermal and sieve cell activity. The consistency between these cell-type activity predictions between the developmental atlas (Woo et al. 2016) and the bolting pseudotime (Redmond et al. 2023) suggests that these developmental shifts in cell activity are robust and confirms that the pseudotime is effectively ordered by a developmental trajectory.

We were curious whether the changes in cell types over time were associated more with the plant's development or with its other traits such as biomass or leaf size. For each cell type, the Spearman's correlation coefficient was calculated between the proportion of that cell type in each plant and that plant's pseudotime, wet biomass, or leaf area (Fig. 4C, Supplementary Fig. S8). The proportion of vascular cells, bundle sheath, and sieve cells were more strongly correlated with biomass than with pseudotime, suggesting that the size of the plant may be associated with water and sugar transport. On the other hand, mesophyll, epidermal, and companion cells were more associated with the pseudotime and and therefore may be more closely associated with development.

**Figure 5.**
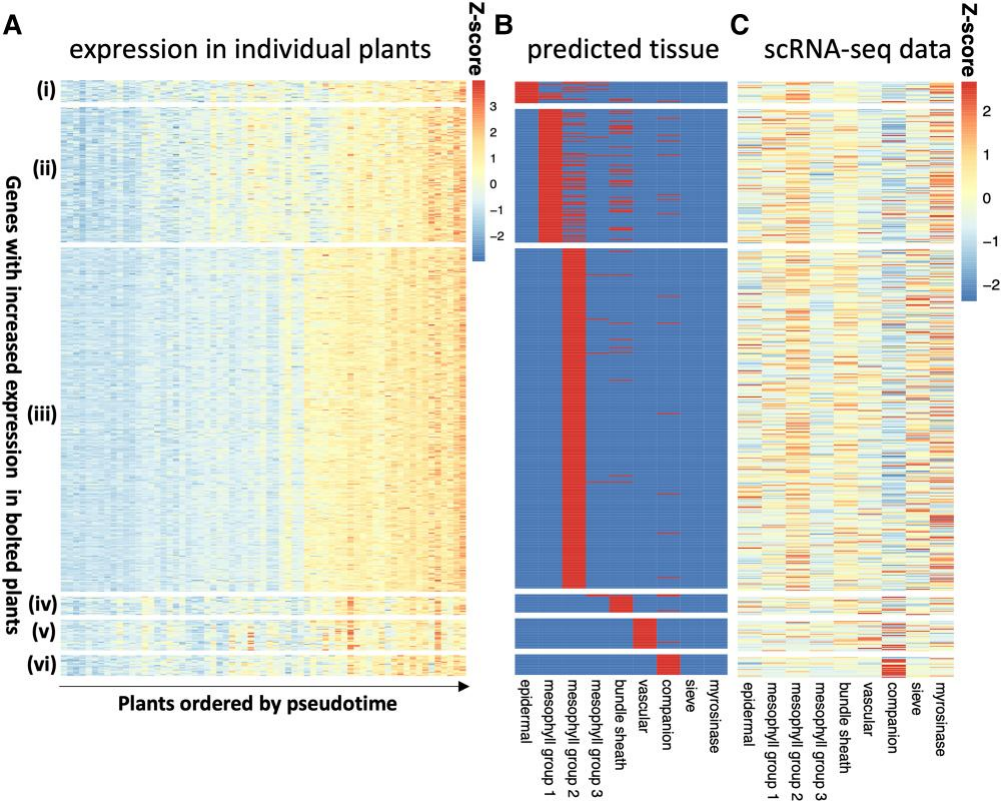
Cell-type-specific gene expression of bolting-related genes. Using the high-resolution imputing function in CIBERSORTx, we predicted cell-type-specific expression of genes that were differentially expressed in bolted/unbolted plants. In this study, we show the results for the genes that have higher expression levels in bolted plants, with the inverse gene set shown in Supplementary Fig. S9. **A)** The *z*-score of the expression of these genes in the bulk RNA-seq experiment (Redmond et al. 2023), grouped by the cell type in which they were predicted to be expressed and ordered by pseudotime. **B)** The cell-type assignment, with red indicating that a gene is expected to be found in that cell type. **C)** The *z*-score of the mean expression of these genes in the scRNA-seq dataset (Procko et al. 2022).

Next, we investigated the cell-type-specific processes taking place during bolting, utilizing the capacity of CIBERSORTx to impute cell-type-specific expression from bulk RNA-seq data. The cell-type-specific genes increased or decreased their expression at different points in the pseudotime using the bulk RNA-seq data from Redmond et al. (2023) (Fig. 5A, Supplementary Fig. S9A), suggesting that at least part of the differences in the timings of expression of genes across pseudotime could be a result of different activity levels of different cell types. Most of the genes were identified as being associated with mesophyll Group 2 (Fig. 5B, Supplementary Fig. S9B). There are some consistencies with the mean expression levels of these genes in the scRNA-seq dataset (Procko et al. 2022), specifically among mesophyll Group 2 cells and companion cells for genes that increase with pseudotime and with mesophyll Group 2/3 and sieve cells for genes that decrease with pseudotime (Fig. 5C, Supplementary Fig. S9C). However, there are also some inconsistencies, such as being unable to reconstitute the epidermal expression pattern. Over all genes, the imputed expressions are relatively consistent with the known gene expression values (Supplementary Fig. S10).

First, we analyzed the GO terms of cell-type-specific genes whose expression increased over pseudotime (Supplementary Table S7). Those associated with mesophyll Group 2 tended to be found in membranes and involved in adenosine triphosphate, guanine triphosphate, and nicotinamide adenine dinucleotide+ binding (GO:0005524, GO:0005096, and GO:0003953) and the regulation of endocytosis (KEGG:04144). Genes found in the epidermis were enriched via vesicle-mediated transport (GO:0016192). Vascular genes were associated with sulfur metabolism (KEGG:00920) and response to nutrient levels (GO:0031669).

Among genes that reduced their expression over pseudotime (Supplementary Table S8), there was enrichment in chloroplast and photosynthesis-related GO terms in epidermal and mesophyll cell types. Epidermal cells were also enriched in porphyrin (a pigment) metabolism (KEGG:00860) and amino acid metabolism–related processes (KEGG:00300). Mesophyll Group 3 was associated with the cell cycle (GO:0007049) and DNA replication initiation (GO:0006270), while both Groups 2 and 3 were enriched for components of the ribosome (GO:0005840) compared with other cell types. We noted that mesophyll Group 3 was also expressed in early development and at night, and therefore, potentially this cell type was in a dividing and growing state.

### AraLeTA: an Arabidopsis Leaf Time-dependent Atlas

**Figure 6.**
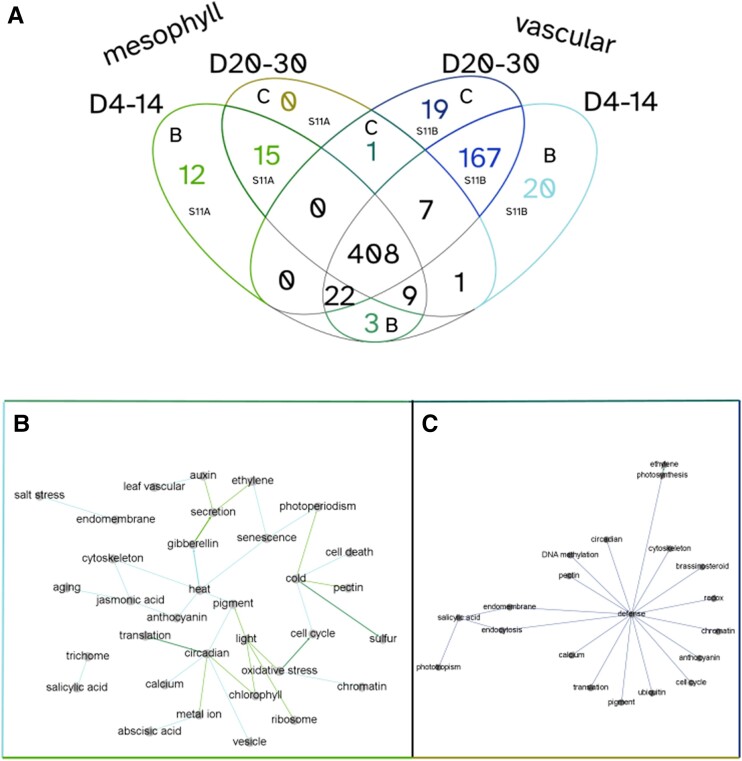
PAFway networks across different tissue types at different ages. Using PAFway, we generated networks of functional terms to assess changes in function between tissue types as they age. **A)** Mesophyll (Groups 1, 2, and 3) and vascular-associated (bundle sheath, phloem, vascular, companion, guard, and sieve) cells showed a large overlap in functional edges across age groups, with some network edges that are unique to specific tissues and ages. **B)** A network of functional terms that are associated with young mesophyll and vascular cells. **C)** A network of functional terms associated with old mesophyll and vascular cells. Together, these show the changing network over time, suggesting that these cells perform different physiological functions as they age. The colors of the edges in **B**) and **C**) correspond to the associated colored sections of the Venn diagram in **A**).

Our previous results have demonstrated that there are different regulatory programs that are active at different times of day, different developmental stages, and different cell types. As a resource for the community, we have developed AraLeTA (Arabidopsis Leaf Time-dependent Atlas; https://regulatorynet.shinyapps.io/araleta/), which can be used to identify the portions of the Arabidopsis gene regulatory network that are active in different contexts, by filtering based on expression values (Fig. 6). As its basis, it utilizes the DNA affinity purification sequencing (DAP-seq) network developed by O’Malley et al. (2016), but it enables the user to filter the network by the age, time of day, and cell types of interest, highlighting edges in which both the source and the target are expressed above a threshold value under the relevant conditions. This kind of thresholding approach uses similar criteria for filtering the gene regulatory network as what was used by Ferrari et al. (2022) and has been shown to enrich the gene network for true edges. The AraLeTA network can be downloaded or visualized as a heatmap or graph.

As it is often difficult to make sense of large “hairball” networks, we have incorporated PAFway additionally, a program for identifying the relationships between functional terms within the topology of the network (Mahjoub and Ezer 2020). This allows us to find patterns in the rewiring of the network over time, within specific cell types.

To highlight the utility of AraLeTA, we focused on 4 conditions: (i) young (4 to 14 d postgermination) mesophyll (Groups 1, 2, and 3) cells, (ii) old (20 to 30 d postgermination) mesophyll cells, (iii) young vascular (bundle sheath, phloem, vascular, companion, guard, and sieve) cells, and (iv) old vascular cells (Fig. 6A). To select thresholds for our networks, we varied the single-cell and bulk RNA-seq threshold parameters and evaluated their impact on the number of significant functional edges (*P* < 0.05) selected by the PAFway network, choosing thresholds that generated either local maxima or inflection points in the size of the PAFway network (Supplementary Fig. S11). As expected, young mesophyll cells included many associations with light response (Fig. 6B). Interestingly, there were more defense-, jasmonic acid–, and salicylic acid (SA)-related edges in vascular cells, reflecting the transport mechanism of these plant hormones (Supplementary Fig. S12, A and B). The old plant networks were centered on SA, a senescence-associated plant hormone, and defense (Fig. 6C). Many of the genes with GO terms related to defense are also involved in senescence, especially in response to necrotrophic pathogens (Woo et al. 2016). Redmond et al. (2023) also demonstrated that processes such as ubiquitination, endocytosis, cell cycle, translation, and response to redox are all perturbed at the onset of senescence, and these terms all appear in the network associated with older plants. These general trends confirm that the filtering criteria are adequately selecting relevant portions of the DAP-seq network.

## Discussion

### Cell-type-specific transcriptional activity: what does it mean?

In this study, we illustrated how we used CIBERSORTx to infer the transcriptional activity of different cell types within a bulk RNA-seq sample. One must be nuanced in our interpretation of what a change in transcriptional activity means from a biological standpoint (Supplementary Fig. S13). When one cell type is predicted to have a higher proportion than another cell type, there are many alternative explanations: (i) there may be a higher proportion of one cell type in the sample relative to the other (Supplementary Fig. S13A). (ii) One cell type may have a higher overall transcription rate than the other (Supplementary Fig. S13B). (iii) There may be only one cell type that fluctuates between transcriptional states. For instance, it is unclear whether it is possible for cells that belong to a particular mesophyll group to transition into a cell from a different mesophyll group (Supplementary Fig. S13C); (iv) cells may express a transcriptional pattern that is similar to other cell types at certain timepoints, and the algorithm may be misassigning the transcriptional activity to these cell-type categories (Supplementary Fig. S13D). To make this latter point more concrete: while most photosynthesis in Arabidopsis takes place in mesophyll cells, other cell types also contain some low density of chloroplasts (Ishikawa et al. 2020). The increased transcriptional activity of photosynthetic processes in these other cell types at certain times of day may lead to an overestimation of mesophyll transcriptional activity by CIBERSORTx, as photosynthesis-related processes are used as markers for mesophyll transcriptional activity. We choose to use the term “cell-type-specific transcriptional activity” to highlight the fact that we are analyzing the relative frequency of cell-type-specific patterns of transcription. Despite the multiple different underlying processes that can lead to changes in cell-type-specific transcriptional activity, as we define here, this concept is still very useful, because it provides us with a summary of the distinct transcriptional programs that are occurring in samples that contain mixes of different cell types.

### Shifts in cell transcriptional activity over different timescales

**Figure 7.**
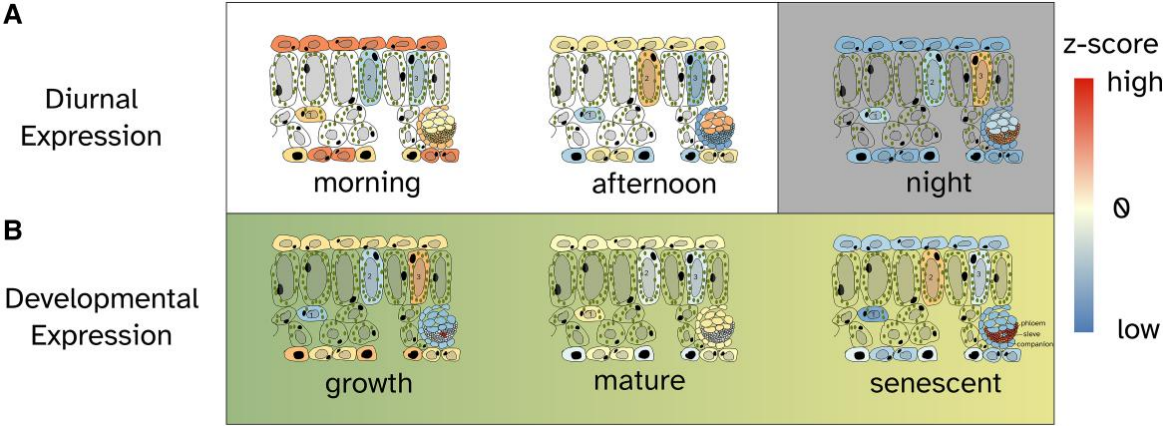
A graphical abstract showing the cell-type-specific expression pattern. This refers to the relative changes in gene expression values across diurnal **A**) and developmental **B**) time series. The colors correspond to the color scale of the heatmaps in Fig. 2.

We show that the transcriptional activity of several different cell types varies both diurnally and developmentally, as summarized in Fig. 7, A and B. This suggests that waves of expression in bulk RNA-seq time series may represent waves of transcriptional activities of different cell types, rather than waves of regulatory activity—an assumption that is often held when inferring gene regulatory networks based on bulk RNA-seq data (Huynh-Thu and Geurts 2018). Time-series single-nucleus RNA-seq (Lee et al. 2023) and spatial transcriptomics (Giacomello 2021) in plants promise to better distinguish temporal and spatial waves.

### Patterns of mesophyll transcriptional states

Single-cell RNA-seq and single-nucleus RNA-seq have consistently identified subclustering of mesophyll cells, both in Arabidopsis and in rice (Kim et al. 2021; Wang et al. 2021; Procko et al. 2022; Tenorio Berrío et al. 2022; Xia et al. 2022). These studies have enabled the identification of cell-type-specific markers for different subgroups of mesophyll; however, the main roles of these subclusters have not been fully characterized, and the mesophyll groups do not have obvious physical differences. Our analysis augments Procko et al.’s (2022) analysis of the 3 mesophyll groups (as summarized in Table 1), by revealing the time in which each group is most likely transcriptionally active, both on a diurnal scale and on a developmental scale.

**Table 1.** A summary of the properties of mesophyll cell types

Mesophyll	Period of development	Time of day	Light-sensitive genes	Impact of bolting on relative expression
1	Mature	Morning	*LNK1, LNK3, STO, LHCB2, LHCA4*	Increase
2	Senescence	Afternoon to early evening		Increase
3	Growth	Night		Decrease
Stressed	Late senescence	2 to 4 h after dawn	*DNAJ, SR45a, MBF1C*	N/A

It is important to remember that different parts of the same individual plant may perceive time in different ways, a form of intraorganismal heterochrony. Gould et al. (2018) found that there are waves of circadian expression that spread spatially from the meristem and root tips to the remainder of the plant, which could result in circadian asynchrony between cells in the leaf under free-running conditions. Asynchrony of the clock may also arise between cells in the leaf due to the entrainment of the clock in the morning through exogenous sugars (Haydon et al. 2013). There is also intraorganismal heterochrony in relation to biological age. Different leaves will be at different stages of development at the same chronological time (Efroni et al. 2008; Redmond et al. 2023). It may even be possible for different parts of the same leaf to have different biological ages at the same chronological age, due to localized stresses. The individual transcriptional profiles sampled using a single-cell or single-nucleus RNA-seq method may represent not only heterogeneous cell types but also heterogeneous biological times. By combining single-cell RNA-seq data with high temporal resolution bulk RNA-seq, we may be able to begin to dissect this kind of temporal heterogeneity too.

### CIBERSORTx enables greater exploitation of RNA-seq datasets

While single-cell RNA-seq is becoming increasingly common in plants, it is still too expensive and cumbersome to perform these experiments over high-resolution time series, under a wide range of environmental conditions and under a wide range of genotypes. In addition, there are tens of thousands of existing bulk RNA-seq datasets available that could vary in their cell-type proportion (Zhang et al. 2020). In this study, we show that plant scRNA-seq datasets can be used to train a CIBERSORTx model that can be used to algorithmically dissect the bulk RNA-seq samples by their cell types. This will confer Arabidopsis researchers who do not have the capacity to do scRNA-seq the ability to exploit these data for enhancing their research. Additionally, this kind of analysis could be used to help decide which timepoints would be most informative for performing single-cell RNA-seq, using a similar computational approach as in Ezer and Keir (2019). Moreover, AraLeTA is a useful platform for utilizing existing bulk RNA-seq time-series data, single-cell RNA-seq data, and transcription factor–binding data to isolate spatial and temporal regulatory processes of interest. Together, CIBERSORTx and AraLeTA provide us with an atlas of leaf expression in Arabidopsis over cell type and over time.

## Materials and methods

### Running CIBERSORTx

Seurat was used to visualize and process the single-cell RNA-seq data (Hao et al. 2021). In the matrix provided to CIBERSORTx (Newman et al. 2019) to generate the signature matrix, genes that were very lowly or very highly expressed were filtered out, with ln(total read count) between 4 and 10. For the purposes of testing CIBERSORTx on the simulated bulk RNA-seq data, a random sample of 75% of the cells was used to generate the signature matrix and a Pearson's correlation, and correlation tests were deployed on the remaining 25% testing set. For the rest of the paper, all cells were used to generate the signature matrix. The cluster designations used in Procko et al. (2022) were used as the phenotype classes. In all cases, we disabled quantile normalization (which is recommended for RNA-seq data) and used 100 runs for the permutation tests. For imputing the gene expressions in each sample in the bolted plants, we used only the 9 most abundant cell types and “other” and focused only on genes that were identified as upregulated or downregulated in bolted/not bolted plants, according to Redmond et al. (2023).

### Generating simulated bulk RNA-seq samples

To simulate bulk RNA-seq samples composed on a single cell type, the remaining cells from Procko et al. (2022) that were not used to generate the model were randomly partitioned into 2 equal groups for each cell type and summed together, forming Replicates 1 and 2 for each cell type. We also simulated bulk RNA-seq samples from the single-cell microdissected samples from Xia et al. (2022). In this case, we summed over the single-cell RNA-seq samples from each cell type associated with a specific leaf.

To simulate the mixed bulk RNA-seq samples, we not only wanted to have somewhat realistic cell-type proportions but also have a wide variation in cell types between samples. For each cell type, we first calculated the proportion of cells of that type in the single-cell RNA-seq dataset. Then, per cell type, we simulated the proportion of cells within the bulk RNA-seq sample according to a normal distribution pattern, with a mean and Sd equal to the cell type proportion within the single-cell RNA-seq dataset. To calculate the final number of cells per cell type, we rounded up any negative values to 0 and multiplied this by the average number of cells we wanted per sample (600). We randomly selected the specified number of cells from each cell type and then calculated the sum of transcript counts per gene for the associated set of cells and considered that to be our simulated bulk RNA-seq sample.

### Bioinformatics analysis

All clustering was performed using the default hierarchical clustering parameters of the pheatmap package (Kolde 2019). GO enrichment analysis was performed using gProfiler (Kolberg et al. 2023). Networks were visualized using Gephi (Bastian et al. 2009) and iGraph (Csárdi et al. 2023). PAFway (Mahjoub and Ezer 2020) was used to generate a graph of the related functional annotations of the basis of the text analysis of the GO Slim annotations provided by Berardini et al. (2015) and the topology of the DAP-seq gene network (O’Malley et al. 2016). AraLeTA was developed as a Shiny App (Chang et al. 2023). The PAFway networks were visualized using Gephi (Bastian et al. 2009). All codes for generating the figures are available at https://github.com/stressedplants/AraletaScripting/. All codes for the Shiny app are available at https://github.com/stressedplants/AraLeTA/.

### Accession numbers

The data underlying this article are available in the NCBI's Gene Expression Omnibus under data libraries under accession numbers GSE43616, GSE182414, and GSE184511 and in the NCBI's Sequence Read Archive under PRJNA668247, PRJNA224133, and PRJNA395645. All TAIR IDs are found in Supplementary Tables S2, S4 to S6.

## Supplementary Material

kiae117_Supplementary_Data

## Supplementary data

The following materials are available in the online version of this article.

**Supplementary Figure S1**. The ability of CIBERSORTx to detect “mock” bulk RNA-seq samples, consisting of a single cell type.

**Supplementary Figure S2**. The ability of CIBERSORTx to predict the relative composition of “mock” bulk RNA-seq samples with mixed tissues.

**Supplementary Figure S3**. The assignment of cell type to microdissected tissues.

**Supplementary Figure S4**. The analysis of a signature matrix.

**Supplementary Figure S5**. Unscaled heatmaps from Fig. 2.

**Supplementary Figure S6**. The impact of methyl jasmonate (MeJA) on cell-type proportions.

**Supplementary Figure S7.** Unscaled heatmaps from Fig. 4.

**Supplementary Figure S8**. Scatterplots underlying Fig. 4C.

**Supplementary Figure S9**. Cell-type-specific gene expression of genes that have low expression in leaves of bolted plants.

**Supplementary Figure S10**. A comparison of predicted gene expression values and imputed values.

**Supplementary Figure S11**. The identification of scRNA and bulk RNA thresholding values.

**Supplementary Figure S12**. A comparison of predicted gene expression values and imputed values.

**Supplementary Figure S13**. Factors influencing transcriptional activity.

**Supplementary Table S1**. Simulated bulk RNA-seq samples.

**Supplementary Table S2**. A signature matrix learned by CIBERSORTx.

**Supplementary Table S3**. A gProfiler GO summary of signature genes.

**Supplementary Table S4.** Predicted transcriptional activities of cell types across developmental scales.

**Supplementary Table S5**. Predicted transcriptional activities of cell types across diurnal scales and with MeJA exposure.

**Supplementary Table S6**. Predicted transcriptional activities of cell types in bolted and unbolted plants.

**Supplementary Table S7**. A gProfiler GO summary of cell-type-specific genes (more expressed in bolting cells).

**Supplementary Table S8**. A gProfiler GO summary of cell-type-specific genes (less expressed in bolting cells).

## Dive Curated Terms

The following phenotypic, genotypic, and functional terms are of significance to the work described in this paper:

ASN1 Gramene: AT3G47340
ASN1 Araport: AT3G47340

DIN10 Gramene: AT5G20250
DIN10 Araport: AT5G20250

phloem AmiGo: PO:0005417

ASPARAGINE CHEBI: CHEBI:22653

ATP CHEBI: CHEBI:15422

LHCA4 Gramene: AT3G47470
LHCA4 Araport: AT3G47470

LNK1 Gramene: AT5G64170
LNK1 Araport: AT5G64170

LNK3 Gramene: AT3G12320
LNK3 Araport: AT3G12320

STO Gramene: AT1G06040
STO Araport: AT1G06040

LHCB2.4 Gramene: AT3G27690
LHCB2.4 Araport: AT3G27690

SR45a Gramene: AT1G07350
SR45a Araport: AT1G07350

MBF1C Gramene: AT3G24500
MBF1C Araport: AT3G24500

SEN1 Gramene: AT4G35770
SEN1 Araport: AT4G35770
