# Supplementary material for: AraLeTA: An Arabidopsis leaf expression atlas across diurnal and developmental scales
Source: Plant Physiol. 2024 Mar 1;195(3):1941–53. doi: 10.1093/plphys/kiae117 (PMC11213249; doi:10.1093/plphys/kiae117)
Supplement: kiae117_Supplementary_Data [file pp.01831.2023-s04.pdf › pp.01831.2023-s04.pdf]

Plant Physiology®

An Arabidopsis leaf expression atlas across diurnal and developmental scales

Gina Vong, Kayla McCarthy, Will Claydon, Seth Davis, Ethan Redmond, and Daphne Ezer

Review Timeline:

Submission Date:	28-Nov-2023
Editorial Decision:	22-Jan-2024
Revision Received:	08-Feb-2024
Accepted:	09-Feb-2024

22-Jan-2024

Dr. Daphne Ezer
University of York
Biology
Heslington YO310RT
United Kingdom

RE: An Arabidopsis leaf expression atlas across diurnal and developmental scales

Dear Dr. Daphne Ezer:

We are pleased to accept your manuscript for publication in the Plant Physiology. This acceptance is contingent on revision based on the comments of our reviewers. In particular, please consider the following:

Both reviewers are enthusiastic about the manuscript, but both have suggestions for minor clarifications. In addition, Reviewer #2 has asked for some additional analysis that would add value to the final version of the manuscript.

Please highlight all changes and include a detailed annotation to changes to the text, with line numbers, and noting your responses to the comments.

To submit your revised manuscript, click:

Link Not Available

If you cannot return the revised manuscript within 8 weeks of receipt, please let us know. Otherwise, we will assume that you have elected not to revise the manuscript and are withdrawing it.

Thank you for allowing us to review your work. We look forward to hearing from you soon.

Sincerely,

Plant Physiology Board of Editors

-- Reviewer comments:
Reviewer #1 (Comments for the Author):

Vong, McCarthy, and Claydon report an atlas of cell type-specific gene expression changes in Arabidopsis leaves over time. Using a published deconvolution technique (CIBERSORTx), the authors train a model on published leaf scRNA-seq data and subsequently infer relative expression of cell types across published time series bulk RNA-seq data. This manuscript is timely and of special interest to the community because high costs currently prohibit most plant labs from generating finely resolved time series scRNA-seq datasets. In addition to new insights about gene expression dynamics in Arabidopsis leaf cells, this manuscript provides a blueprint for plant researchers to synthesize new information from published data. Overall, this work is a step toward key community goals such as profiling the responses of major organ systems to environmental changes at single cell resolution (Ahmed et al., 2021 eLife).

The manuscript is well written and the logic is easy to follow. I have only a few minor suggestions.

Line 95: Could the authors elaborate on the selection criteria for the two single cell RNA-seq datasets (Procko et al and Xia et al) chosen for this study? For example, did the authors consider the quality of the data and the cell type annotations, the number of cells profiled, and/or the quality of the metadata (e.g., were growth and experimental conditions described in detail)? Including these details will provide an example of scRNA-seq data analysis and management considerations for the community, including authors of future scRNA-seq publications and those who will choose published data to analyze.

Line 105: Could the authors provide a brief description of why they chose CIBERSORTx? Are there benchmarking studies of similar algorithms applied to mammalian data?

Line 447: The authors make a clear case for the current cost-effectiveness of inferring cell type transcriptional activities from bulk RNA-seq data. However, I am curious about the long-term outlook. As costs for single cell experiments (hopefully) decrease over time, do the authors envision scenarios where researchers might choose to re-analyze bulk datasets with techniques such

as CIBERSORTx instead of or before performing a time series single cell experiment? Maybe for pilot studies to help choose the most informative time points, genotypes, or treatments for a single cell experiment?

AraLeTA: The browser is easy to navigate and nicely laid out. I suggest adding a legend to the bulk and single cell RNA-seq filtering threshold widgets to indicate what the units are.

Reviewer #2 (Comments for the Author):

In the manuscript "An Arabidopsis leaf expression atlas across diurnal and developmental scales," Vong et al. describe the use of the computational tool CIBERSORTx on Arabidopsis leaf transcriptomic datasets. Previously untested using plant transcriptomic datasets, CIBERSORTx enables the deconvolution of bulk RNA sequencing datasets into the relative cellular activities of individual cell types following training with a comparable single cell transcriptomic dataset. Using a leaf single cell atlas (Procko et al., 2022) the authors document the successful application of CIBERSORTx in the prediction of cellular activity of individual cell types with training datasets and extend the use of the CIBERSORTx to various bulk Arabidopsis transcriptomic datasets that more precisely follow developmental and diurnal regulation of Arabidopsis leaves.
Interestingly, a secondary analysis of published bulk transcriptome datasets using the trained CIBERSORTx revealed previously undefined distinctions in cell type specific transcriptional activity based on both diurnal and developmental contexts, both between distinct cell types (mesophyll vs vascular/phloem/companion cells) and also within individual cell types (subpopulations of mesophyll cells). As noted by the authors, there is a wealth of publicly available transcriptomic datasets that can be further analyzed using deconvolution tools such as CIBERSORTx to determine cell type specific activity as additional single cell and single nuclei transcriptomic datasets from Arabidopsis tissues become available.
Overall, the manuscript is well written, and the figures are presented clearly and understandably. The authors have also generated an online interface to access and query the outputs of the dataset, which will be of interest to the community. Below are some minor comments that may help to improve the manuscript.

It is interesting that the "cellular activity," as in the relative transcript abundance that originates from an individual cell type, can be highly dynamic throughout development and diurnal rhythms. The authors note that the relative activity of individual cell types are altered during growth transitions (lines 152-160), including epidermal and mesophyll cells, as well as with the onset of senescence (lines 290-292). Can this finding be corroborated in the original single cell dataset when quantifying the number of transcripts per cell in clusters corresponding to young cells vs. senescent and/or stressed cells of the same cell type?

Along the same lines, the CIBERSORTx deconvoluted mature rosette dataset (Fig2A) appears to have the highest relative cellular activity of mesophyll group 1, bundle sheath, vascular, and epidermal cell types. Is that trend also observable in the single cell dataset?

It might be useful to include the original dimensionality reduction and clustering results from Procko et al., 2022 for convenient viewing within the article (combined with Figure S1?). The numbering and annotation of various clusters can be somewhat arbitrary based on the original dataset (mesophyll 1, 2, 3; unknown 1, 2, 3). For example, based on the original cluster results, cluster 10, renamed unknown group 1, is intermediary between the mesophyll and epidermis clusters and may help to support the idea of cell type transitions and/or transitory cell states as discussed on Lines 163-166.

Figure S1 legend seems to be out of date - The figure legend describes two figures (A) and (B) but there is only one figure present

Line 194: typo in the citation of Gould et al., 2018

Line 436: I believe the authors intended "entrainment" of the clock

Figure S8A, Y-axis is incorrectly labeled

Figure 4C: The correlative analysis between cell type activity and growth metrics is interesting. I would also be interested in viewing the correlative analyses in a figure that was also able to depict the individual data points of each of the 65 individual plants

Line 320: I believe the text is referencing Fig S9

The text in Figure S11A is low resolution and not easily legible

--- Reviewer comments:
Reviewer #1 (Comments for the Author):

Vong, McCarthy, and Claydon report an atlas of cell type-specific gene expression changes in Arabidopsis leaves over time. Using a published deconvolution technique (CIBERSORTx), the authors train a model on published leaf scRNA-seq data and subsequently infer relative expression of cell types across published time series bulk RNA-seq data. This manuscript is timely and of special interest to the community because high costs currently prohibit most plant labs from generating finely resolved time series scRNA-seq datasets. In addition to new insights about gene expression dynamics in Arabidopsis leaf cells, this manuscript provides a blueprint for plant researchers to synthesize new information from published data. Overall, this work is a step toward key community goals such as profiling the responses of major organ systems to environmental changes at single cell resolution (Ahmed et al., 2021 eLife).

The manuscript is well written and the logic is easy to follow. I have only a few minor suggestions.

Line 95: Could the authors elaborate on the selection criteria for the two single cell RNA-seq datasets (Procko et al and Xia et al) chosen for this study? For example, did the authors consider the quality of the data and the cell type annotations, the number of cells profiled, and/or the quality of the metadata (e.g., were growth and experimental conditions described in detail)? Including these details will provide an example of scRNA-seq data analysis and management considerations for the community, including authors of future scRNA-seq publications and those who will choose published data to analyze.

This is an excellent suggestion. We added the following sentence on line 95:
'We chose to use Procko et al. (2022) because it had a larger number of leaf cells than many other single cell datasets, so we were able to include rarer cell types. Moreover, the authors did not artificially enrich their selection of specific cell types, so the ratio of cell types would be roughly similar to that found in a natural leaf.'

On line 109 we added:
'We chose to test our model on cells in Xia et al., (2022) as these were isolated using microdissection, so we would be able to know the spatial localisation of these cells. In contrast, in marker-based selection, there is always a risk that the marker is also expressed in an off-target cell type.'

Line 105: Could the authors provide a brief description of why they chose CIBERSORTx? Are there benchmarking studies of similar algorithms applied to mammalian data?
We actually already reference the major mammalian benchmark study in the introduction on line 69-71, but now we include the words 'benchmarking study' to make it clear that this is the kind of study that was performed:

'Drawing inspiration from cancer cell dynamics research (Newman et al., 2019), we 70 used CIBERSORTx, a powerful technique that combines single-cell RNA-seq and bulk RNA71 seq data (Newman et al., 2019) which outperforms other deconvolution methods in a large benchmarking study (Sutton et al., 72 2022).

Line 447: The authors make a clear case for the current cost-effectiveness of inferring cell type transcriptional activities from bulk RNA-seq data. However, I am curious about the long-term outlook. As costs for single cell experiments (hopefully) decrease over time, do the authors envision scenarios where researchers might choose to re-analyze bulk datasets with techniques such as CIBERSORTx instead of or before performing a time series single cell

experiment? Maybe for pilot studies to help choose the most informative time points, genotypes, or treatments for a single cell experiment?

This is a great idea. In fact, we have already developed a different computational tool for identifying informative time points from time series data, which could take the output of CIBERSORTx as an input. We incorporated this suggestion in the discussion on line 454:

'Moreover, this kind of analysis could be used to help decide which time points would be most informative for performing single cell RNA-seq, using a similar computational approach as in Ezer and Keir et al (2019).'

AraLeTA: The browser is easy to navigate and nicely laid out. I suggest adding a legend to the bulk and single cell RNA-seq filtering threshold widgets to indicate what the units are.

This has been added and is a great suggestion.

Reviewer #2 (Comments for the Author):

In the manuscript "An Arabidopsis leaf expression atlas across diurnal and developmental scales," Vong et al. describe the use of the computational tool CIBERSORTx on Arabidopsis leaf transcriptomic datasets. Previously untested using plant transcriptomic datasets, CIBERSORTx enables the deconvolution of bulk RNA sequencing datasets into the relative cellular activities of individual cell types following training with a comparable single cell transcriptomic dataset. Using a leaf single cell atlas (Procko et al., 2022) the authors document the successful application of CIBERSORTx in the prediction of cellular activity of individual cell types with training datasets and extend the use of the CIBERSORTx to various bulk Arabidopsis transcriptomic datasets that more precisely follow developmental and diurnal regulation of Arabidopsis leaves.
Interestingly, a secondary analysis of published bulk transcriptome datasets using the trained CIBERSORTx revealed previously undefined distinctions in cell type specific transcriptional activity based on both diurnal and developmental contexts, both between distinct cell types (mesophyll vs vascular/phloem/companion cells) and also within individual cell types (subpopulations of mesophyll cells). As noted by the authors, there is a wealth of publicly available transcriptomic datasets that can be further analyzed using deconvolution tools such as CIBERSORTx to determine cell type specific activity as additional single cell and single nuclei transcriptomic datasets from Arabidopsis tissues become available.
Overall, the manuscript is well written, and the figures are presented clearly and understandably. The authors have also generated an online interface to access and query the outputs of the dataset, which will be of interest to the community. Below are some minor comments that may help to improve the manuscript.

It is interesting that the "cellular activity," as in the relative transcript abundance that originates from an individual cell type, can be highly dynamic throughout development and diurnal rhythms. The authors note that the relative activity of individual cell types are altered during growth transitions (lines 152-160), including epidermal and mesophyll cells, as well as with the onset of senescence (lines 290-292). Can this finding be corroborated in the original single cell dataset when quantifying the number of transcripts per cell in clusters corresponding to young cells vs. senescent and/or stressed cells of the same cell type?

For clarity, we address this suggestion in tandem with the suggestion below.

Along the same lines, the CIBERSORTx deconvoluted mature rosette dataset (Fig2A) appears to have the highest relative cellular activity of mesophyll group 1, bundle sheath, vascular, and epidermal cell types. Is that trend also observable in the single cell dataset?

The original dataset only contained only single cells from a single time point (17 days) as specified on line 177 of the manuscript. Therefore, it is impossible to directly corroborate these findings with the same single cell data set that was used to train the CIBERSORTx model. However, we have now included a more thorough comparison with an additional dataset that has an Arabidopsis developmental single cell atlas (Lee et al. 2023). Our analysis and this dataset are not directly comparable, because whole organisms are used instead of just leaves. Nevertheless, we agree with the reviewer that it is useful to state where our results agree/disagree with this data set.

We have added the following lines in 166:
'Although there is currently no leaf-specific developmental single cell atlas to compare our results to, Lee et al, 2023 performed a whole rosette single cell RNA-seq in three developmental timepoints. Both this dataset and CIBERSORTx show a decline in bundle sheath and vascular cells from day 17 onwards. Lee et al. (2023) showed that the proportion of epidermal cells stays stable, as the plant transitions to senescence, suggesting that our observed decline in epidermal cells may reflect a change in transcriptional activity, rather than a decline in cell count. Our results contradict Lee et al., in terms of phloem parenchyma and companion cell expression, which we predict increase and Lee et al., predict decline. These discrepancies may come from the activity of phloem parenchyma cells in non-leaf tissue in the Lee et al. (2023) dataset, as it includes whole rosettes. Moreover, Lee et al. (2023) appeared to have a larger proportion of senescing cell types in day 17 rosettes than at older ages, so the sampled rosettes at this time point may have been stressed, making direct comparisons challenging.'

It might be useful to include the original dimensionality reduction and clustering results from Procko et al., 2022 for convenient viewing within the article (combined with Figure S1?). The numbering and annotation of various clusters can be somewhat arbitrary based on the original dataset (mesophyll 1, 2, 3; unknown 1, 2, 3). For example, based on the original cluster results, cluster 10, renamed unknown group 1, is intermediary between the mesophyll and epidermis clusters and may help to support the idea of cell type transitions and/or transitory cell states as discussed on Lines 163-166.

This has been done. The revised label of Figure S1 now reads:
'**Figure S1: Ability of CIBERSORTx to detect 'mock' bulk RNA-seq samples, consisting of a single cell type.** (A) We were successfully able to replicate the UMAP and cluster designations in Procko et al. (2022), demonstrating the reproducibility of that work. From the original Procko manuscript, we renamed clusters 1, 3, 4 as mesophyll groups 1, 2, 3; and unknown clusters 10, 15, 16 as unknown groups 1, 2, 3, for clarity. (B) CIBERSORTx was trained on 75% of the cells from each of these clusters. The remaining cells were used to develop simulated bulk RNA-seq samples. For each cluster, we generated two simulated bulk RNA-seq samples of pure tissue, by summing the expression of 50% of the cells from that cluster that were not used for training. The heatmap shows the proportion of each cell type (columns) that was predicted for each simulated bulk RNA-seq sample (rows). The simulated RNA-seq samples are labelled by the cell type that was used to generate the simulated bulk RNA-seq sample.'

Figure S1 legend seems to be out of date - The figure legend describes two figures (A) and (B) but there is only one figure present

This has been done, in conjunction with the addition of the suggested Figure S1A above.

Line 194: typo in the citation of Gould et al., 2018

This has been fixed.

Line 436: I believe the authors intended "entrainment" of the clock

This has been corrected.

Figure S8A, Y-axis is incorrectly labeled

This has been corrected.

Figure 4C: The correlative analysis between cell type activity and growth metrics is interesting. I would also be interested in viewing the correlative analyses in a figure that was also able to depict the individual data points of each of the 65 individual plants

We added these scatterplots as Supplementary figure S6. We actually find them very compelling additions, as they illustrate the strength of some of the correlations so clearly.

Line 320: I believe the text is referencing Fig S9

This has been corrected. Indeed, we realised that we had skipped our reference for S6 in the text, so the references to Figures S7 onwards were off by one. This has been fixed.

The text in Figure S11A is low resolution and not easily legible

This has been corrected.

09-Feb-2024
Dr. Daphne Ezer
University of York
Biology
Heslington YO310RT
United Kingdom

MSID: PP2023-RA-01831R1
MS TITLE: An Arabidopsis leaf expression atlas across diurnal and developmental scales

Dear Dr. Daphne Ezer:

I am pleased to inform you that your revised manuscript "An Arabidopsis leaf expression atlas across diurnal and developmental scales" has been accepted for publication in Plant Physiology. Your manuscript will be checked for consistency with journal formatting and image standards by one of our Science Editors, who will contact you shortly. They may have a number of queries and suggestions for you. Please keep in mind that they work to increase the online visibility of your article and its attraction for readers from the widest of backgrounds. You will have the opportunity to review any revisions, and I hope you will work constructively with them to get the most out of your research. You will also have the opportunity to review all changes when you receive the author proofs for your article.

Once validated for publication, your article will be sent to a Science Editor for a final review of your manuscript and source files. After your final files are uploaded, your article will appear in the next available issue and the online Preview version of your article will be posted after your signed Copyright and Checklist forms and final source files are received.

Please be sure to include the following in your manuscript file:
Harvard style citations
List of all author contributions and funding information at the beginning of manuscript after the titles and author list.
List all supplemental materials associated with the article, with titles, at the end of the ms, before the references and acknowledgments section.
Complete Figure Legends for all figures appearing in the article listed before the Literature Cited.

Thank you and congratulations on your Plant Physiology paper!

Sincerely,
Plant Phys Board of Editors

=====
FIRST AUTHOR PROFILES
=====

We recognize the hard work that goes into being First Author of a Plant Physiology paper by publishing a "First Author Feature" for each First Author alongside your paper.

If the first author(s) would like to submit information for a feature on Facebook and Twitter, fill out the form by clicking on the link below:

Plant Physiology First Author Feature Form: https://forms.office.com/r/ZBCGZfhm8F
If you cannot access the form through the link above, please contact Rachel at rbelsky@aspb.org.

To upload an image of yourself to accompany your profile, please put your photo in the correct journal folder at the link below. You must label your image: Author First Name Last Name Article Title.

First Author Profile Image Dropbox: https://www.dropbox.com/scl/fo/xz029p7qjr2jml2rp2kmq/h?dl=0&rlkey=6fkxcaqmxx35ap152b2osy1fz

=====

IMPORTANT REMINDER: PEER REVIEW REPORTS
=====

If you opted to publish a peer review report along with your article during the original submission process, it will be prepared by the editorial staff and publicly posted with your manuscript, inside the zip file that contains any other supplemental material. As a reminder, the peer review report is a public record of all comments from editors and reviewers, as well as your prior responses, as you received them in the decision letters for each draft of your manuscript. If you agreed to publish this report and have changed your mind, or are not sure if you selected this option, please contact the editorial office as soon as possible before signing the license agreement from our publisher.

--- Reviewer comments:
